# Effect of intracutaneous pyonex therapy on postoperative pain management following perianal surgery: A systematic review and meta-analysis

**DOI:** 10.1371/journal.pone.0296439

**Published:** 2024-01-19

**Authors:** Ning Xu, Kailian Jiang, Lulu Liu, Xiao Yang

**Affiliations:** 1 Department of Anesthesiology, Weihai Central Hospital Affiliated to Qingdao University, Weihai City, Shandong Province, China; 2 Department of Respiratory & Critical Care Medicine, Weihai Central Hospital Affiliated to Qingdao University, Weihai City, Shandong Province, China; Charles Darwin University, AUSTRALIA

## Abstract

Intracutaneous pyonex therapy (IPT), a novel acupuncture technique also known as intradermal thumbtack needle embedding therapy, has been reported to optimize postoperative pain management following perianal surgery. This meta-analysis aimed to analyze the efficacy of IPT for postoperative pain management following perianal surgery. The Cochrane Library, PubMed, EMBASE, Web of Science, CNKI, SinoMed, Wanfang, and VIP databases were systematically searched for randomized controlled trials (RCTs) on IPT as a treatment for postoperative pain management following perianal surgery from inception until June 15, 2022. The analyzed outcomes from the eleven RCTs included in this meta-analysis were as follows: postoperative visual analogue scale(VAS), analgesic duration, ineffective cases following treatment, and adverse events. Subgroup analyses were conducted according to different time points. Risk-of-bias assessment, publication bias analysis, sensitivity analysis, and trial sequential analysis were performed. Of the 895 patients, 450 and 445 were included in the IPT and control groups, respectively. The IPT group showed a better analgesic effect[standard mean difference (SMD) = –0.77, 95% CI: –1.00 to –0.53, *P* < 0.00001; *P* for heterogeneity = 0.009, I^2^ = 59%] and longer analgesic duration [SMD = 0.56, 95% CI: 0.31 to 0.82, *P* < 0.0001; *P* for heterogeneity = 0.6, I^2^ = 0%], fewer ineffective cases following treatment [risk ratio(RR) = 0.23; 95% CI: 0.13 to 0.39, *P* < 0.00001; *P* for heterogeneity = 0.76, I^2^ = 0%], and lower overall occurrence of postoperative complications [RR = 0.35; 95% CI: 0.17 to 0.70; *P* = 0.003; *P* for heterogeneity = 0.85, I^2^ = 0%] than the control group. Thus, our findings indicated that IPT can provide better pain management following perianal surgery compared to controls. This novel approach complements a reasonable modality for postoperative multimodal analgesia and is worth promoting.

## Introduction

Perianal disorders are common worldwide and are characterized by structural and functional dysfunctions [1]. Patients with perianal disorders seek treatment including surgical interventions for aggravated symptoms [2]. Most patients experience mild to moderate pain during the perioperative period of perianal surgery, whereas some experience severe postoperative pain [3, 4]. Postoperative pain is not only a common cause of delayed discharge but also one of the leading reasons for readmission of patients after perianal surgery [5, 6]. Appropriate postoperative pain management for such patients is critical, but remains problematic [5]. Multimodal analgesia methods facilitate postoperative pain management [7] and are currently used to optimize postoperative pain management after perianal surgery, including patient-controlled intravenous analgesia (PCIA), oral administration of analgesic drugs, and acupuncture [8–10]. Among the various analgesic methods, traditional acupuncture, which consists of various acupuncture techniques, has been increasingly used as an integrative or complementary therapy for pain. Acupuncture is well tolerated with little risk of serious adverse effects [11, 12]. Recently, a novel acupuncture technique called intracutaneous pyonex therapy (IPT) has been reported to be effective as a non-pharmacological postoperative analgesic regimen that complements the currently prevalent concept of multimodal analgesia.

IPT is a novel acupuncture technique that uses a different acupuncture apparatus than traditional acupuncture, and is also perceived as an intradermal thumbtack needle embedding therapy. The SEIRIN ^®^ brand press-pins, manufactured by Japan’s Qingling Co., Ltd. and commonly used in this therapy, are shaped like thumbtacks and are also called pyonex or thumbtack needles. Different needle models (0.2 mm × 1.2–1.8 mm) are selected according to the body type of the patients [13, 14]. IPT is performed by intracutaneously inserting the needle into specific acupoints for a set period and applying intermittent pressure to the needle handle. The needle continually and consistently stimulates skin nerve endings, regulates the body’s *qi* and blood, balances *yin* and *yang*, dredges meridians, and regulates organs, thus treating the disease [8, 15]. IPT is a relatively simple procedure with a more acceptable needle sensation than traditional acupuncture therapy [8]. IPT has recently been used in various fields, including analgesia and sedation in critically ill patients receiving mechanical ventilation [14], postoperative pain management after perianal surgery [16], functional nasal endoscopy [15], laparoscopic cholecystectomy [17], and hip arthroplasty [18]. Furthermore, previous research has shown that IPT is a safe and effective method of analgesia that should be promoted in patients undergoing perianal surgery [8]. However, systematic evidence regarding the use of IPT for postoperative pain management after perianal surgery is lacking. Our meta-analysis aimed to determine whether IPT could optimize postoperative pain management after perianal surgery.

## Materials and methods

### Pre-registration and study design

The systematic review protocol for the current study was registered in the International Platform of Registered Systematic Review and Meta-Analysis Protocols (registration number: INPLASY202260046; DOI number: 10.37766/inplasy2022.6.0046). The present meta-analysis adhered to the recommendations of the Preferred Reporting Items for Systematic Reviews and Meta-Analyses (PRISMA) statement [19] and the guidelines described in the Cochrane Handbook. The PRISMA checklist 2020 [20] is provided as supplement (S1 Checklist).

### Information sources and search strategy

We searched electronic databases, including the Cochrane Library, PubMed, EMBASE, Web of Science, SinoMed, CKNI, WanFang, and VIP, from inception until June 15, 2022. Published randomized controlled clinical trials (RCTs) documenting the use of IPT as a treatment for postoperative pain management following perianal surgery were collected. The search keywords included pyonex, thumb-tack needle, postoperative pain, and perianal surgery.

### Eligibility criteria

The following aspects were the inclusion criteria for this meta-analysis:

1. Participants: We identified adult patients who required surgical treatment for perianal disorders (excluding tumors), such as hemorrhoids and anal fistulas.
2. Interventions: Participants were divided into two groups (IPT and control) based on whether IPT was used for postoperative pain management.
3. Outcome measurement: The postoperative outcomes analyzed included the visual analogue scale (VAS) score at different time points, analgesic duration (the period between the disappearance of pain after treatment and its reappearance), ineffective cases after treatment (no improvement in pain symptoms after treatment), and complications.
4. Study type: Only RCTs were included.

The exclusion criteria were as follows: (i) traditional acupuncture techniques other than IPT; (ii) failure to provide original data; (iii) duplicate literature, similar reports, or incomplete information; (iv) literature type discrepancy; and (v) poor-quality literature reports.

#### Study selection and data extraction

Study selection and data extraction were independently conducted by two authors (K. L. J. and N. X.). The identified studies were imported into Endnote X9 to duplicate. Then, the titles and abstracts of the remaining studies after excluding duplicates were carefully evaluated against the eligibility criteria. Subsequently, those ineligible studies were excluded. Full-text reviews for all the included studies were conducted when necessary. A PRISMA flowchart was created to demonstrate the literature screening process. A data extraction table covering the baseline information of the included studies, methodological characteristics, routine details of the study population, and specific information on the treatment and outcomes for both groups was prepared in advance. Two authors independently extracted the requested data from the eligible studies. The third author (X. Y.) resolved disagreements regarding the process of study selection and data extraction.

### Risk of bias assessment

Two authors separately used the Cochrane Risk of Bias Assessment Tool to assess the risk of bias in the eligible studies [21]. The following were investigated: random sequence generation, allocation concealment, blinding of participants and personnel, blinding of outcome assessment, incomplete outcome data, selective reporting, and other biases. Review Manager, version 5.4 (Cochrane Collaboration, Oxford, UK) was used to evaluate the details of the risk of bias in eligible studies. The results of risk-of-bias assessment were ranked as “low,” “unclear,” and “high.”

### GRADE certainty assessment

The Grading Recommendations Assessment, Development, and Evaluation (GRADE) approach was used to assess the quality of evidence for the incorporated outcomes [22]. Illustrative comparative risks, number of studies, certainty evaluation, relative effects, quality of evidence, and importance were the main criteria employed for the GRADE certainty assessment. Furthermore, the certainty evaluation included study design, risk of bias, inconsistency, indirectness, imprecision, and other considerations. The GRADEprofiler (version 3.6.1; GRADE Working Group, EU) was used to generate a table depicting the overall results of the GRADE certainty assessment.

### Statistical analysis

Statistical analysis of the included studies was performed using the Review Manager 5.4 software. For continuous variables, the related data were represented as standardized mean difference (SMD) and 95% confidence interval (CI). For dichotomous variables, the related data were calculated as pooled risk ratio (RR) and 95% CI. The results were considered statistically significant when the *P*-value was < 0.05. Heterogeneity among the included studies was analyzed using I^2^ statistics. An I^2^ statistic of <50% was not considered to indicate obvious heterogeneity, and a fixed-effects model was adopted. Conversely, a random-effects model was used. Based on different time points, a subgroup analysis was performed on the postoperative VAS score. A sensitivity analysis was performed if the I^2^ statistic was >50%. Egger’s test was performed using Stata 15.1 software to calculate the *P*-value for publication bias analysis, and *P*>0.05 showed no significant publication bias.

### Trial sequential analysis (TSA)

TSA software version 0.9.5.10 Beta (Center for Clinical Intervention Research, Copenhagen, Denmark) was used to estimate the sample size and evaluate the possibility of random errors in this meta-analysis. The required information size (RIS) value was counted using TSA software. Trials with no adverse events were included in the analysis. A fixed-effects model analysis was performed and we set α (Type-I error) = 0.05, β (Type-II error) = 0.2 (1-β = 80% power), and a relative risk reduction = 20% to cumulate the Z-curve and generate the boundaries of conventional and TSA. To conclude the final TSA results, we mainly focused on whether the sample size reached the RIS and whether the Z-curve crossed the conventional and TSA boundaries. Finally, the TSA software was used to generate a figure of the adjusted boundary prints depicted in this study.

## Results

### Search results

**Fig 1:**
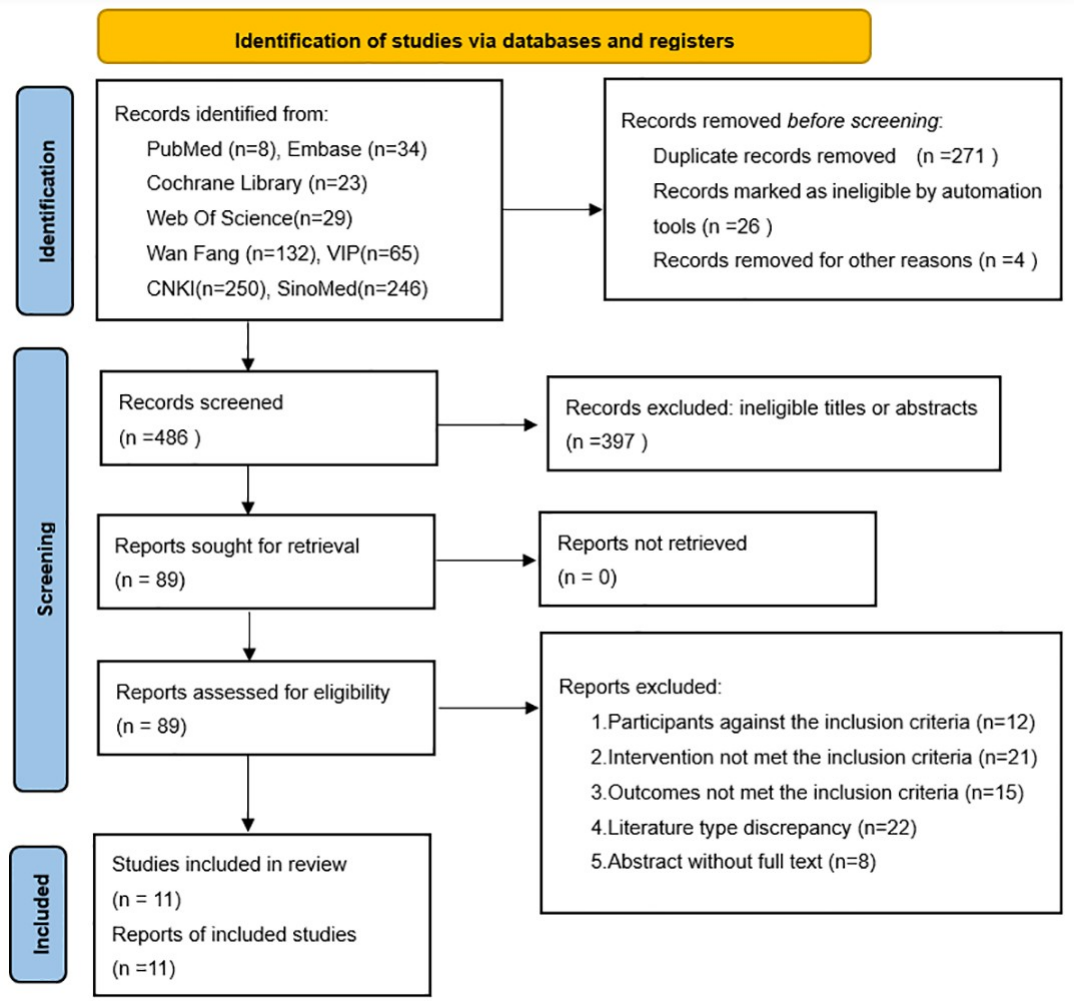
Diagram of the literature search and screening process.

The selection and screening of the literature yielded 787 relevant articles, which were imported into the Endnote X9 software. 301 records were removed due to duplication, ineligibility marked by automation tools and other reasons. Then, the titles and abstracts of the remaining studies were carefully screened. Subsequently, 397 studies with ineligible titles or abstracts were excluded, and 89 studies were assessed for eligibility against the inclusion criteria. After excluding studies that did not report eligible participants, interventions, outcomes, literature type, or text completeness, we eventually included 11 RCTs in the meta-analysis. Fig 1 illustrates the literature search and screening processes.

### Characteristics of the incorporated studies

Table 1 presents the characteristics of the studies included. Eleven randomized controlled trials met the eligibility criteria. All of these studies were published in Chinese and included 895 patients, with 450 and 445 patients in the IPT and control groups, respectively. Perianal disorders include anal fissures, fistulas, hemorrhoids, and mixed hemorrhoids. Eleven acupoints of IPT were incorporated, and the acupoint most frequently embedded by pyonex was Erbai (*EX-UE2*). Analgesic methods comparable to IPT include oral analgesics, PCIA, and traditional intervention methods.

**Table 1:** Characteristics of the incorporated studies.

Study	Sample, IPT/Control, n	Age, years		Surgical type	Random method	IPT	Comparison	Outcomes
		IPT group	Control group			Acupoints selection		
**Yuan, et al. (2021)** [23]	30/30	39.03±17.19	39.23±17.83	Hemorrhoidectomy	RU	Shangliao (*BL31*), Ciliao (*BL32*), Zhongliao (*BL33*), and Xialiao (*BL34*)	Potassium permanganate hip bath	**I, III**
**Zhang, et al. (2020)** [24]	30/30	36.5±4.8	37.2±4.6	Perianal surgery^1^	RNT	Bilateral Chengshan (*BL57*) and Erbai (*EX-UE2*)	Intravenous drip flurbiprofen axetil injection	**I**
**Zu, et al. (2019)** [25]	60/60	48.91±4.19	48.97±4.16	Perianal surgery^1^	H	Bilateral Erbai (*EX-UE2*)	Oral ketorolac tromethamine capsules	**I, III**
**Wang (2018)** [26]	30/30	49.16±10.53	49.11±10.68	External and internal ligation of mixed hemorrhoids	RNT	Bilateral Erbai (*EX-UE2*)	Thunderfire moxibustion^2^	**I, III**
**Lee et al. 2021** [27]	40/40	43.03±9.95	42.53±10.60	External and internal ligation of mixed hemorrhoids	RU	Changqiang (*GV1*), Chengshan (*BL57*), and Erbai (*EX-UE2*)	None	**IV**
**Ye, Ye, and Wang (2022)** [28]	85/80	50.03±10.68	49.21±10.55	Automatic ligation of mixed hemorrhoids	RU	Bilateral Erbai (*EX-UE2*)	Oral celecoxib capsules	**II**
**Guo (2021)** [29]	30/30	29.19±2.84	29.40±2.73	Hemorrhoidectomy	RU	Changqiang (*GV1*)	Traditional intervention methods in clinical practice^3^	**III**
**Yang, and Xu (2021)** [30]	40/40	48.27±6.13	48.32±6.09	Perianal surgery^1^	M	Hegu (*LI4*), Neiguan (*PC6*), and Zusanli (*ST36*)	Dezocine intramuscular injection	**III**
**Zheng, Meng, and Miao (2020)** [31]	30/30	41.3±13.0	46.9±11.6	Hemorrhoidectomy	RNT	Hegu (*LI4*), Neiguan (*PC6*), and Kongzui (*LU6*)	PCIA	**III, IV**
**Zeng and Li (2020)** [32]	40/40	40±15	39±14	Hemorrhoidectomy	RU	Erbai (*EX-UE2*)	Potassium permanganate hip bath	**III**
**Gao, Cheng, and Lu (2022)** [33]	35/35	63.8±11.6	62.89±12.4	Anal fistula cutting	RNT	Bilateral Ciliao (*BL32*) and Erbai (*EX-UE2*)	Intravenous drip dezocine	**I, IV**

**Notes:** I, postoperative VAS score; II, postoperative analgesic duration; III, postoperative ineffective cases after treatment; IV, postoperative adverse events.

IPT, intracutaneous pyonex therapy; RNT, random number table method; RU, mentioned random with no details; M, Matching gender, age, and disease type between groups; H, Hospital admission order; PCIA, patient-controlled intravenous analgesia pump.

^1^, Perianal disorders included anal fissure, anal fistula, and mixed hemorrhoids;

^2^, Traditional Chinese medicine of analgesia;

^3^, Eat reasonably, keep the wound surface clean, change medication regularly, and encourage patients to get out of bed.

### Risk of bias assessment

**Fig 2:**
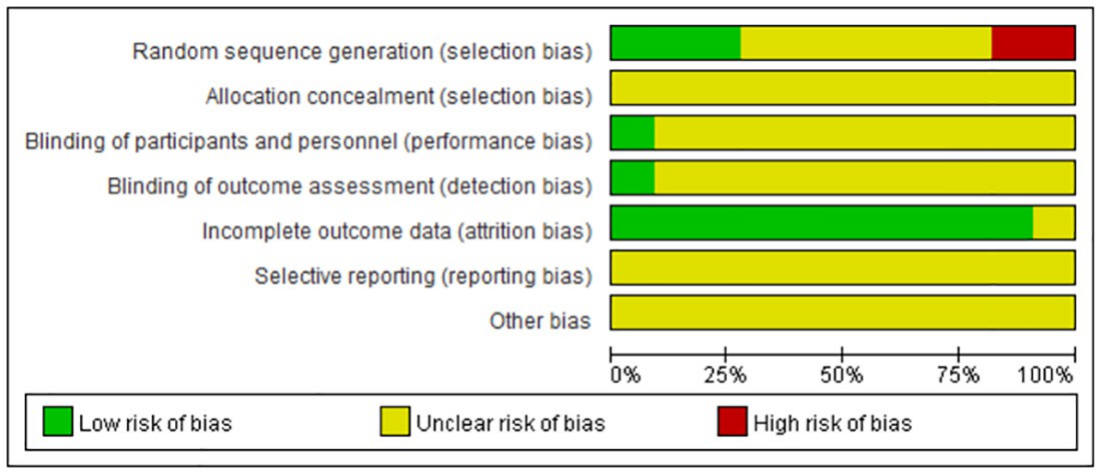
Risk-of-bias summary.

**Fig 3:**
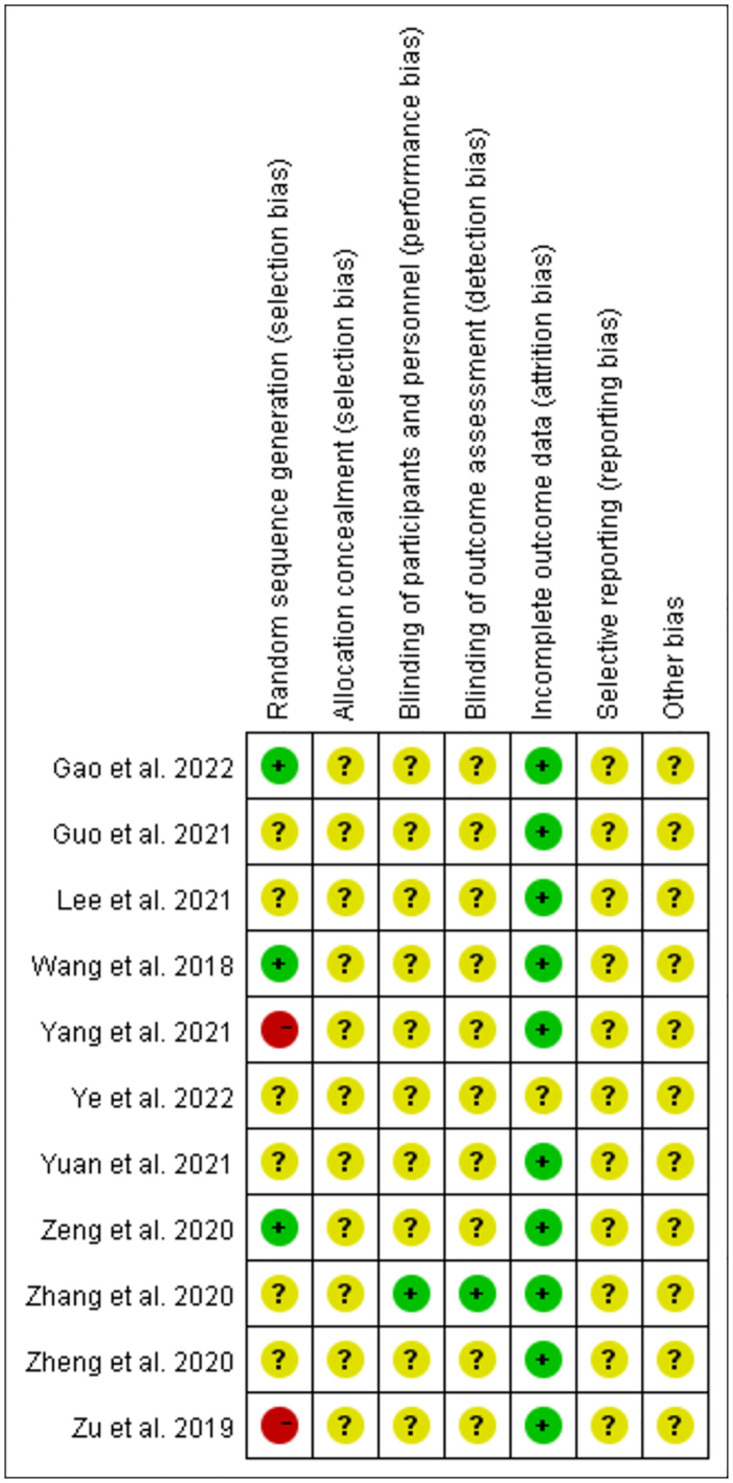
Risk-of-bias graph.

Five randomized controlled trials reported specific methods for random sequence generation, but two had a high risk of bias in their randomization details. Six RCTs mentioned randomization but provided no further details. Most of the included studies had a low risk of bias owing to incomplete outcome data. Most studies covered five types of unclear bias: selection bias of allocation concealment, performance bias, detection bias, reporting bias, and other biases. Figs 2 and 3 present a comprehensive summary of the risk of bias.

### Results of this meta-analysis

#### Postoperative VAS score

**Fig 4:**
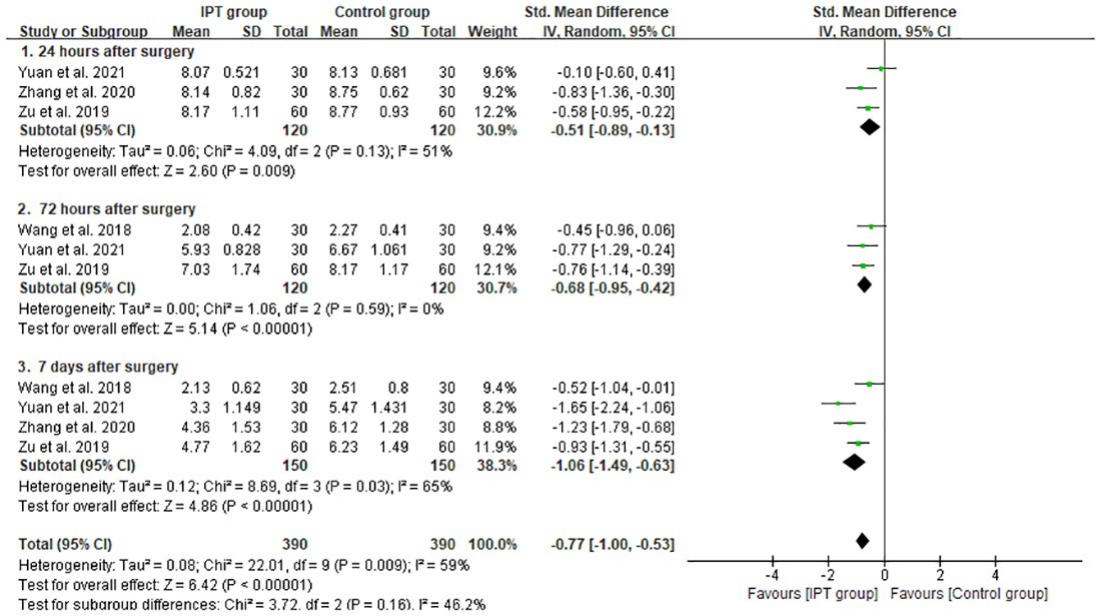
Comparison of forest plot of postoperative VAS score between the IPT and control groups.

Subgroup analysis was conducted at three different time points after surgery (Fig 4). Our meta-analysis indicated a statistically significant difference between the IPT and control groups at 24 h [SMD = –0.51, 95% CI: –0.89 to –0.313, *P* = 0.009; *P* for heterogeneity = 0.13, I^2^ = 51%], 72 h [SMD = –0.68, 95% CI: –0.95 to –0.42, *P* < 0.000001; *P* for heterogeneity = 0.59, I^2^ = 0%] and 7 days [SMD = –1.06, 95% CI: –1.49 to –0.63, *P* < 0.00001; *P* for heterogeneity = 0.03, I^2^ = 65%] after surgery. The IPT group showed a better analgesic effect than the control group in both the early and late postoperative periods.

#### Postoperative analgesic duration

**Fig 5:**
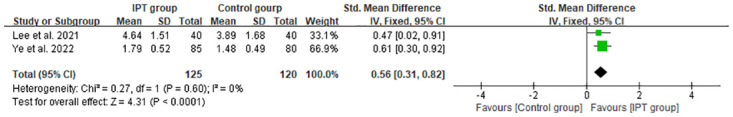
Comparison of forest plot of postoperative analgesic duration between the IPT and control groups.

Two studies [27, 28] documented postoperative analgesic duration (Fig 5). A statistically significant difference was observed between the IPT and control groups [SMD = 0.56, 95% CI: 0.31 to 0.82, *P* < 0.0001; *P* for heterogeneity = 0.60, I^2^ = 0%]. Postoperative analgesic duration was longer in the IPT group than in the control group.

#### Postoperative ineffective cases after treatment

**Fig 6:**
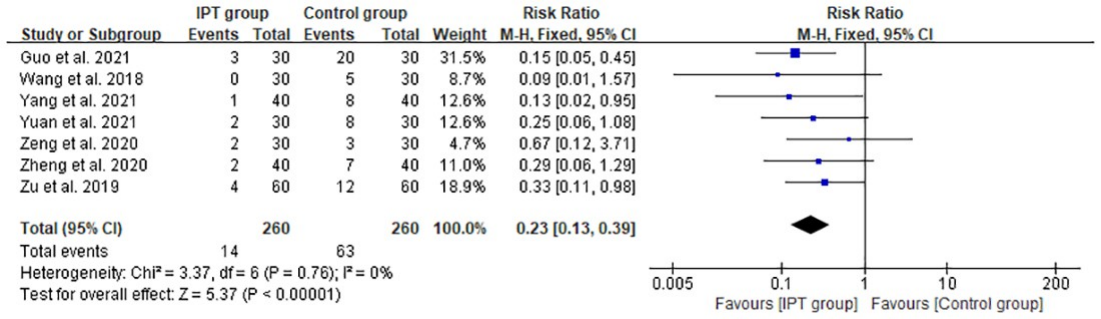
Comparison of forest plot of postoperative ineffective cases after treatment between the IPT and control groups.

Seven studies [23, 25, 26, 29–32] reported the incidence of ineffective cases after treatment (Fig 6). Analysis of the forest plot showed a significant difference between the two groups [RR = 0.23, 95% CI: 0.13 to 0.39, *P* < 0.00001; *P* for heterogeneity = 0.76, I^2^ = 0%]. The pain symptoms of more patients undergoing perianal surgery were relieved with IPT than without IPT(control group).

#### Postoperative complications

**Fig 7:**
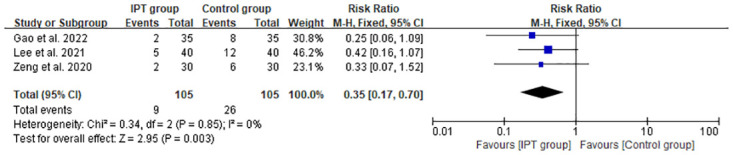
Comparison of forest plot of postoperative complications between the IPT and control groups.

Three studies [27, 32, 33] reported the overall incidence of postoperative complications (Fig 7). The results of the current meta-analysis identified that the IPT group had a lower overall incidence of postoperative complications than the control group [RR = 0.35, 95% CI: 0.17 to 0.61, *P* = 0.003; *P* for heterogeneity = 0.85, I^2^ = 0%].

### Sensitivity analysis

Two subgroups of postoperative VAS score in our meta-analysis showed heterogeneity (I^2^ > 50%). Subsequently, we performed a sensitivity analysis. Excluding the study by Yuan *et al*., which was likely the source of high heterogeneity in the VAS subgroups at 24 h and 7 days after surgery [23], significantly reduced the heterogeneity in these two subgroups.

### GRADE certainty assessment

We followed the GRADE approach to assess the certainty of the evidence for all outcomes using GRADEprofiler software. For the included studies with a high risk of bias in terms of postoperative VAS score and ineffective cases after treatment, the risk of bias was downgraded to “very serious.” For the included studies with unclear risk of bias in terms of postoperative complications, the risk of bias was downgraded to “serious.” For studies wherein high heterogeneity existed in the domains of postoperative VAS score, the inconsistency was downgraded to “serious.” Serious indirectness or imprecision were not detected in any of the four domains. Owing to the large magnitude of the effect (RR < 0.5), the domains of postoperative ineffective cases after treatment and complications were considered to be strongly associated. The GRADE certainty assessment for postoperative ineffective cases after treatment and complications was moderate. Nevertheless, the certainty of the postoperative VAS score and analgesic duration were very low. Table 2 shows the GRADE certainty assessment results.

**Table 2:** Summary of GRADE evidence certainty evaluation.

Patient or population: Adult patients following perianal surgery.													
Intervention: Intracutaneous pyonex therapy.													
Outcome	Illustrative Comparative Risks*(95%)		No of Participants (studies)	Certainty Evaluation						Relative effect (95%CI)			
Assumed risk Control	Corresponding risk IPT		Study design	Risk of bias	Inconsistency	Indirectness	Imprecision	Other consideration		Comments	Quality of the evidence	Importance
**Postoperative ineffective cases after treatment**	**Study population** **242 per 1000** **Moderate** **200 per 1000**	**Study population** **56 per 1000** (31 to 94) **Moderate** **46 per 1000** (26 to 78)	**520** **(7 studies)**	**randomised trials**	**very serious** ^1^	**no serious**	**no serious**	**no serious**	**strong association** ^2^	**RR 0.23** **(0.13 to 0.39)**	**-**	**⊕⊕⊕⊝** **MODERATE**	**CRITICAL**
**Postoperative complications**	**Study population** **248 per 1000** **Moderate** **229 per 1000**	**Study population** **87 per 1000** (42 to 173) **Moderate** **80 per 1000** (39 to 160)	**210** **(3 studies)**	**randomised trials**	**serious** ^3^	**no serious**	**no serious**	**very serious** ^4^	**strong association** ^2^	**RR 0.35** **(0.17 to 0.7)**	**-**	**⊕⊕⊕⊝** **MODERATE**	**IMPORTANT**
**Postoperative VAS score**	**-**	**The mean postoperative VAS score in the IPT group was** **0.69 standard deviations lower** **(0.88 to 0.5 lower)**	**720** **(4 studies)**	**randomised trials**	**very serious** ^1^	**serious** ^5^	**no serious**	**no serious**	**none**	**-**	**SMD -0.69 (-0.88 to -0.5)**	**⊕⊝⊝⊝** **VERY LOW**	**IMPORTANT**
**Postoperative analgesic duration**	**-**	**The mean postoperative analgesic duration in the ITP group was** **0.56 standard deviations higher** **(0.31 to 0.82 higher)**	**245** **(2 studies)**	**randomised trials**	**no serious**	**no serious**	**no serious**	**very serious** ^4^	**none**	**-**	**SMD 0.56 (0.31 to 0.82)**	**⊕⊝⊝⊝** **VERY LOW**	**NOT IMPORTANT**

Notes:

*The basis for the assumed risk (e.g. the median control group risk across studies) is provided in footnotes. The corresponding risk (and its 95% confidence interval) is based on the assumed risk in the comparison group and the relative effect of the intervention (and its 95% CI).

CI, Confidence Interval; CI, confidence interval; RR, relative risk;IPT, intracutaneous pyonex therapy.

^1^. The included studies with a high risk of bias;

^2^. A large magnitude of effect (RR < 0.5);

^3^. The included studies with unclear risk of bias;

^4^. Small sample size and few incidences;

^5^. High heterogeneity existed in this domain.

### Publication bias

**Fig 8:**
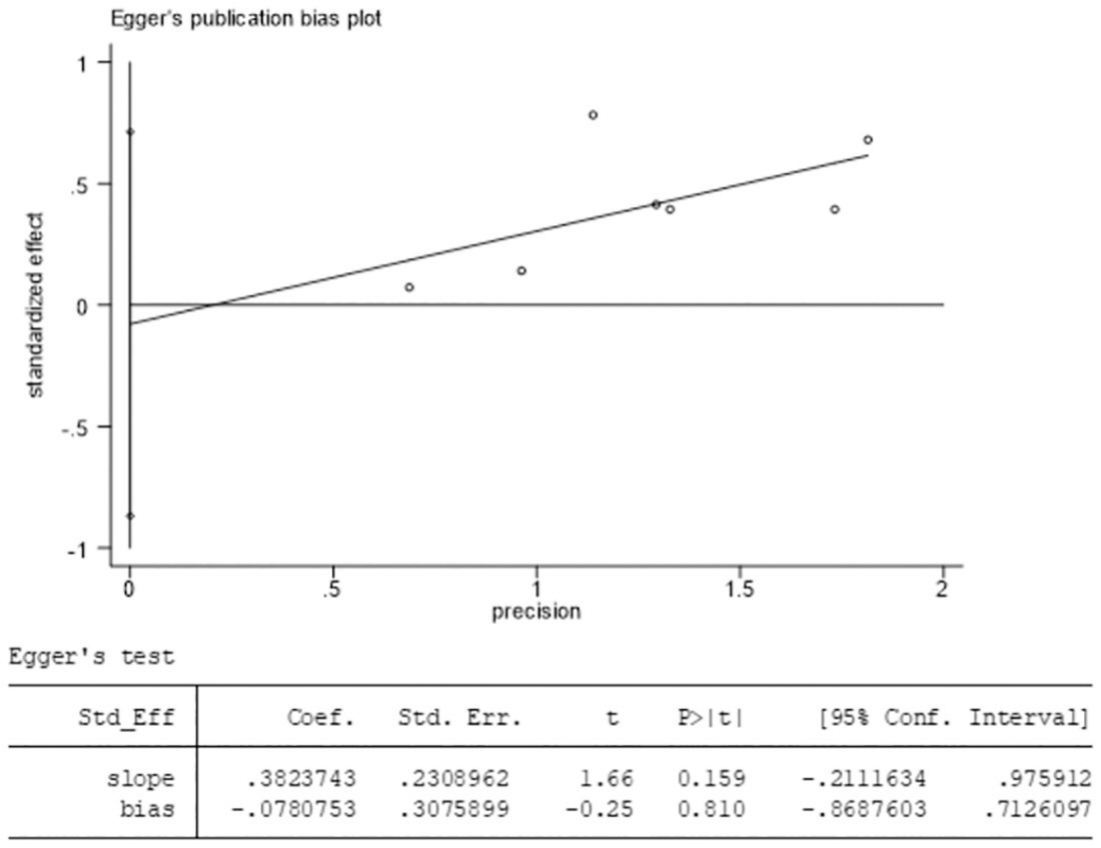
Result of Egger’s test to evaluate postoperative ineffective cases after treatment.

Egger’s test was performed on the included studies to evaluate postoperative ineffective cases after treatment using Stata 15.1 software, and *P*-value was calculated to detect publication bias. The results showed a *P* value of 0.810 (*P* > 0.05), indicating no significant publication bias (Fig 8).

### TSA

**Fig 9:**
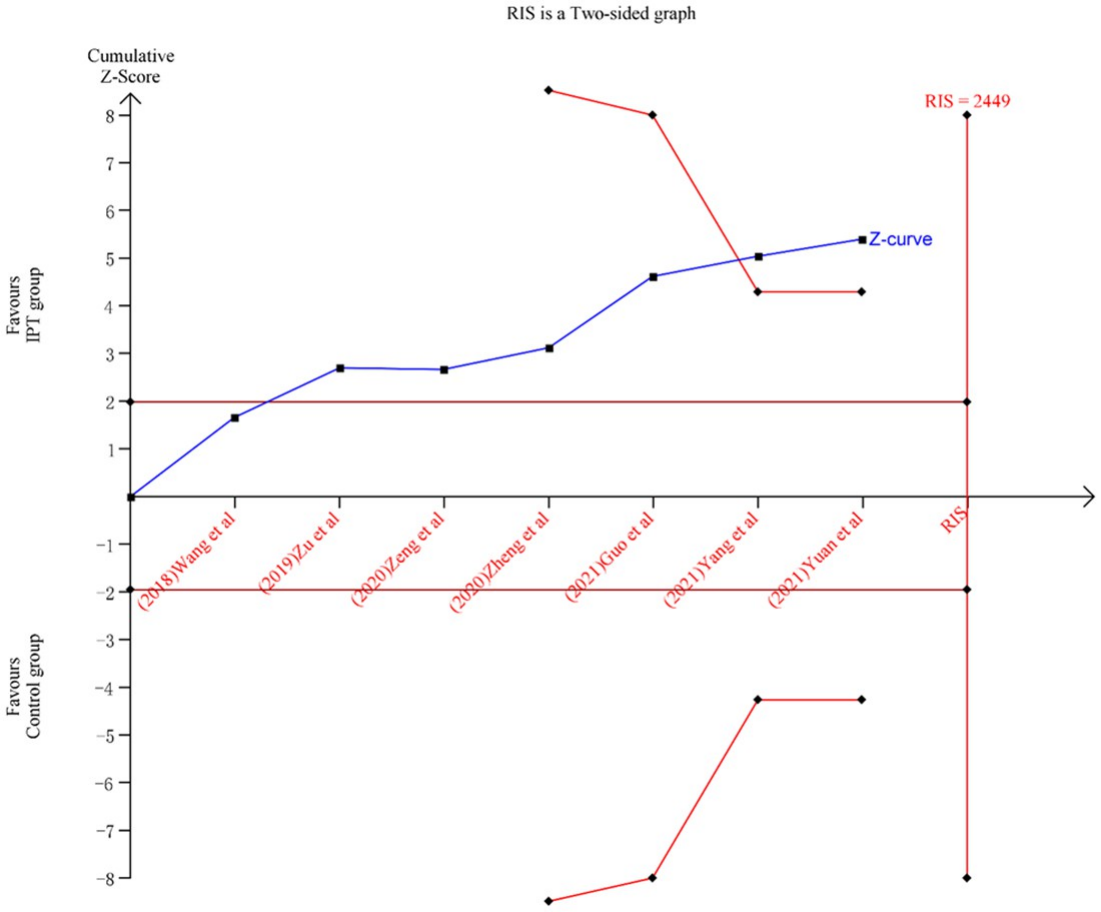
Trial sequential analysis of postoperative ineffective cases after treatment. RIS: required information size.

We conducted a TSA to test the cumulative effect on postoperative ineffective cases after treatment. When considering the RIS of this outcome, we used a control event proportion stemming from the results of this meta-analysis and a relative risk reduction of the incidence of 20%. After performing the required calculations for postoperative ineffective cases after treatment (Fig 9), we found that the Z-curve crossed the conventional and TSA boundaries. However, the sample size for the primary outcome did not exceed the RIS. Based on the above findings, IPT is concluded in advance to be more effective in postoperative pain management after perianal surgery compared to controls. Considering the quality of the included studies, more high-quality studies are required to further validate the results.

## Discussion

We conducted a meta-analysis of 11 studies to test the efficacy and safety of IPT and found that IPT was effective in postoperative pain management after perianal surgery, as indicated by reduced postoperative VAS score, prolonged postoperative analgesic duration, and reduced incidence of ineffective cases after treatment and postoperative complications. GRADE certainty assessment ranged from very low to moderate.

IPT, a novel analgesic therapy after perianal surgery, requires careful and multifaceted assessment of postoperative analgesic effects to optimize postoperative pain management. Thus, the analgesic effects of IPT were analyzed in three domains: postoperative VAS score, analgesic duration, and incidence of ineffective cases after treatment. The meta-analysis results showed that postoperative VAS score was reduced after IPT intervention. Previous research [24, 25] has shown that IPT in patients undergoing perianal surgery can reduce pain at various time points after surgery and is of high clinical utility. Subgroup analyses showed that the postoperative VAS score was reduced in both the early and late postoperative periods. Notably, we found high heterogeneity between the two subgroups (VAS score at 24 h and 7 days after surgery). Possible explanations for this high heterogeneity are as follows: first, the assessment modalities adopted after IPT treatment were not uniform. The duration of needle retention and acupuncture point selection was not discussed in detail, and no uniform criteria for IPT implementation were developed. Second, the methodological quality, literature quality, experimental design, and analgesic protocols compared with IPT varied among the included trials. However, the issue of high heterogeneity was resolved by identifying its source [23] through sensitivity analysis. According to traditional Chinese medicine, IPT can induce a favorable analgesic effect and effectively improve local postoperative meridian blockage and poor blood flow by stimulating acupuncture points, promoting blood circulation, unblocking meridians, and activating blood luck [24, 34]. In modern medicine, the IPT mechanism consists primarily of a weak and persistent stimulation of peripheral nerve receptors and central nerves, suppression of pathological excitation, and improvement of organism responsiveness [34]. Some studies [35, 36] have found that patients subjected to postoperative IPT treatment had lower levels of inflammatory factors like high sensitivity C-reactive protein, interleukin-6, interleukin-17, and tumor necrosis factor-α. IPT inhibits inflammatory responses and exerts analgesic effects. In addition, we included two trials to assess the effect of IPT on analgesic duration. Our meta-analysis demonstrated that IPT prolonged analgesic duration in patients undergoing perianal surgery. Our findings are consistent with those of previous studies [37], showing that IPT is a long-lasting therapy. IPT implementation provides a longer duration of pain relief and significant improvement in the patient’s symptoms, which helps speed up recovery after surgery [28].

Furthermore, we tested seven studies that reported incidence of ineffective cases after treatment [23, 25, 26, 29–32]. According to this meta-analysis, following IPT treatment, pain symptoms in more patients undergoing perianal surgery were relieved. Further, although TSA indicated that the sample size of this domain did not reach the required information size, we could conclude in advance that IPT is more effective for postoperative pain management after perianal surgery compared to controls. Although the incidence of ineffective cases after treatment was lower in the IPT group than in the control group, all the literature in this domain reported ineffective treatment, implying that the analgesic efficacy of IPT has some limitations. According to a prospective cohort study, a multimodal analgesic approach consisting of local anesthetic application, multiple oral analgesics, and written information seemed ineffective in providing adequate pain management after perianal surgery [38]. Pain management after perianal surgery is challenging; therefore, we recommend that more research on the combined use of IPT and various analgesic modalities be conducted, as multimodal analgesia based on IPT combination therapies may have a more positive effect on postoperative pain management after perianal surgery.

Finally, we included three RCTs to analyze the effect of IPT on postoperative complications. The results revealed that the IPT group had a lower incidence of overall postoperative complications than the control group, which is in accordance with previous findings [31, 32]. Postoperative complications, such as postoperative nausea and vomiting, dizziness, headache, pruritus, chest tightness, and shortness of breath, have been reported in the literature enrolled in this meta-analysis [27, 33]; however, we did not conduct a subgroup analysis based on various types of complications owing to insufficient data. It is still not clear whether these complications are caused by surgery or the IPT. Further studies are needed to confirm the safety of IPT according to the different types of postoperative complications and to clarify whether these complications originate from surgery or the IPT.

This meta-analysis had a few limitations. The most important limitation was the inferior quality of the enrolled studies, as most areas were not within a low risk of bias. Second, no uniform standard for the selection of IPT implementation or acupuncture points with a large range of controls based on various analgesic regimens was found in the enrolled studies, resulting in a certain risk of bias. Moreover, culture bias may not be overlooked since IPT is not widely utilized or reported in clinical practice when compared with traditional acupuncture. Finally, we did not conduct a subgroup analysis based on the various types of complications owing to insufficient data.

A few noteworthy implications for future research are as follows: (a) Given the inferior quality of the included literature, future high-quality studies are needed to further validate the efficacy and safety of IPT in postoperative pain management after perianal surgery. (b)We recommend that more research be conducted on the combined use of IPT and various analgesic modalities, as multimodal analgesia based on IPT combination therapies may have a more positive effect on postoperative pain management after perianal surgery. (c) The enrolled participants were mostly in their 40s; this is important, especially since patients undergoing perianal surgery are usually older. Further research related to IPT on pain score, analgesic duration, complications, and recovery in an older population should be conducted. (d) IPT is not widely utilized or reported in the clinical practice of pain medicine, and future studies are suggested to develop uniform criteria for IPT implementation to standardize this type of pain intervention research.(e) Further studies are needed to confirm the safety of IPT according to the different types of postoperative complications and to clarify whether these complications originate from surgery or IPT.

## Conclusion

IPT can provide better pain management after perianal surgery compared to controls. This novel approach complements a reasonable modality for postoperative multimodal analgesia and is worth promoting. However, given the limitations of the current evidence, high-quality multicenter RCTs are still required to validate the efficacy and safety of IPT.

## Supporting information

S1 Checklist PRISMA checklist 2020. (DOCX)
